# Efficient modulation of subwavelength focusing via meta-aperture-based plasmonic lens for multifunction applications

**DOI:** 10.1038/s41598-018-31860-1

**Published:** 2018-09-11

**Authors:** Kai-Hao Chang, Yen-Chun Chen, Wen-Hao Chang, Po-Tsung Lee

**Affiliations:** 1 Department of Photonics, College of Electrical and Computer Engineering, National Chiao Tung University, Hsinchu, 300 Taiwan; 2 Department of Electrophysics, College of Science, National Chiao Tung University, Hsinchu, 300 Taiwan

## Abstract

Subwavelength focusing is crucial for many applications in photonics including super-resolution micro/nanoscopy, nanolithography, and optical trapping. However, most nanostructures exhibit poor ability to modulate focusing spot, which makes them hard to achieve ultra-small resolution. Here, we propose three kinds of plasmonic lens (PL) by utilizing different meta-aperture designs for efficient subwavelength focusing modulation. The shape of nanoaperture strongly influences the diffraction properties. Spatial modulation of focusing spot by employing a circular array of proposed nanoapertures is explored. The best focusing performance among these PLs is the design of T-shape nanoaperture, which has great resolution achieving ultra-small focusing spot of 0.14 λ^2^ and 0.20 λ^2^ (λ = 633 nm) for simulation and experiment respectively, better than lots of focusing devices especially by using linear polarization. Multiple-object trapping can be realized by using T-shape nanoaperture-based PL. Our designed PLs with different nanoapertures demonstrate the capability to broaden and integrate different functionalities for on-chip nanotechnologies development.

## Introduction

Plasmonic lens (PL) can serve as a platform for light and matter interaction at subwavelength nanoscale. With different designs of PLs, a variety of light manipulation can be achieved, such as focusing^1,2^, inward and outward surface plasmon wave propagation^3^, beam shaping^4^, and spatial light modulation^5^. Therefore, light can be precisely controlled or guided in a certain optical path. These techniques can be utilized for concentrator of nanolithography^6^, collector of photodector^7^, illuminator of single photon source^8^, integration with 2D-material as modulator^9^, surface excitation of tip-enhanced Raman spectroscopy^10^, and manipulation of nanoparticles^11^.

The control of focusing spot or non-focusing beam for the applications mentioned above is critical and can be implemented by various plasmonic devices. Subwavelength focusing, one of the most discussed subject, is beneficial for imaging system and lithography. However, only few methods can be used to control the focusing and its modulation but in an inefficiently way^12,13^. Most results show that only the lens size can cause large variation of focus but with appearance of additional side effects like shift of focal length and aberration^14^. Furthermore, it is more difficult to modulate the focal spot at the subwavelength scale. Designs for effective beam shaping of subwavelength focusing are crucial and should be developed to meet the requirements for nanophotonics nowadays.

With the development of meta-surface, the freedom of phase modulation is broadened enormously. Light can be manipulated for spin momentum resolving, polarization conversion, wavefront shaping, and compress sensing. By varying the structural design of meta-surface, the radiative light of plasmon modes projects different ratios of x and y-components of electric field. This property can be the solution for efficient tuning of subwavelength focusing. Although various meta-units have been applied for beaming effect, but not been explored for the modulation of subwavelength focusing.

Here, we propose three kinds of nanoapertures as meta-unit for the design of PL with ultra-small focal spot. The dynamic tuning of focal profile can be achieved under different polarizations. The strategies for nanoaperture selection are investigated and discussed. This work also provides the perspective of fundamental diffraction properties for subwavelength nanoaperture, which has been studied only in simple circle aperture without any shape-relative information^15^. Based on these developed knowledge, multifunction applications including beam shaping, polarization conversion, and nano-object manipulation can be realized by our designed meta-apertures.

## Results and Discussion

### Modulation of subwavelength focusing

**Figure 1:**
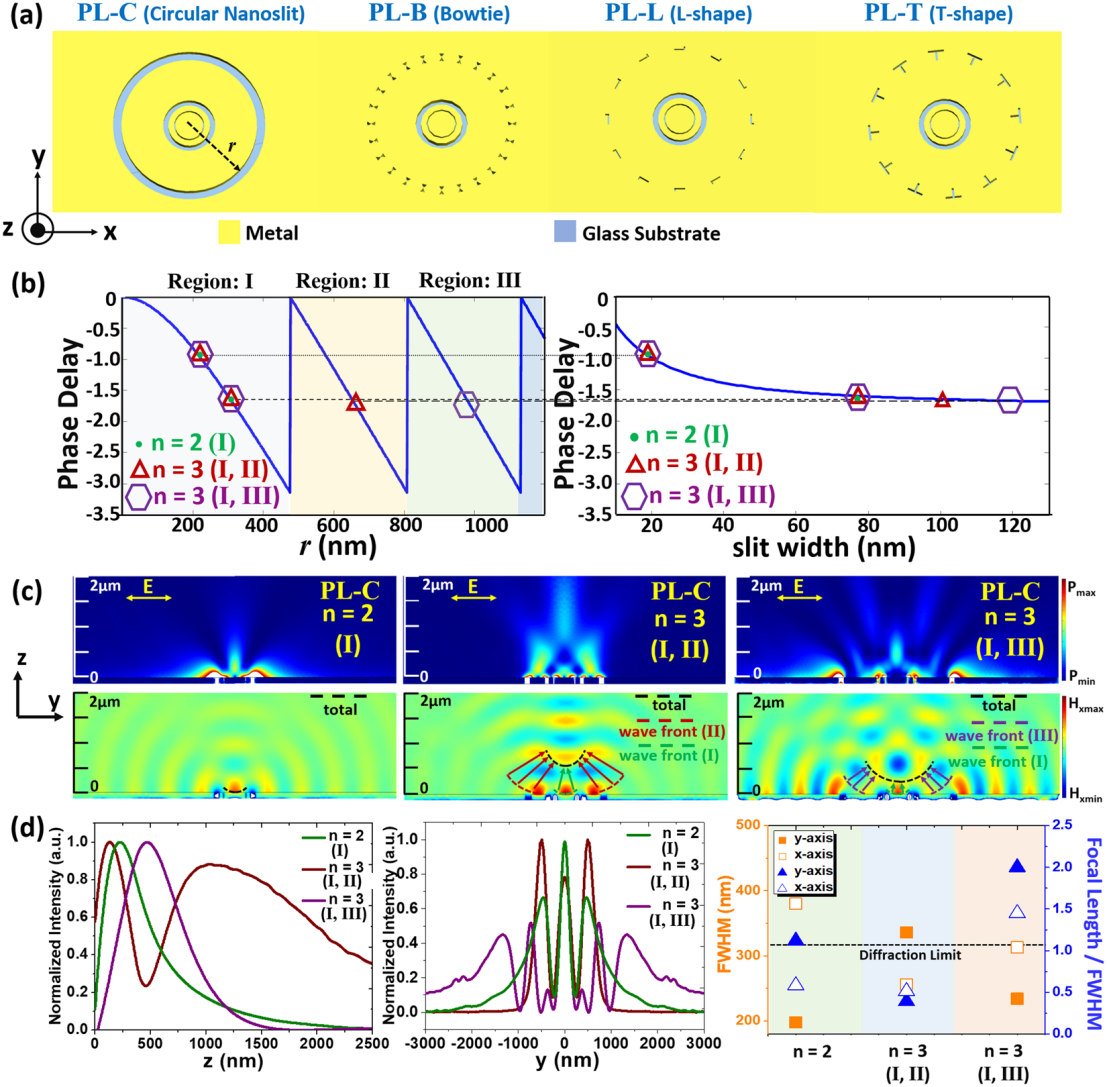
(**a**) Illustration of reference PL with circular nanoslit (PL-C) and proposed meta-aperture-based PLs with bowtie, L, and T shapes (PL-B, PL-L, and PL-T). (**b**) Phase delay of focusing wavefront as a function of radial position *r* for PL-C. Right plot shows the relation between phase delay and slit width. The circle, triangle, and hexagon symbols represent three different designs of PL-C. (**c**) Simulated power intensity (top) and H_x_-field (bottom) distributions of PL-C under linear polarization. The dashed lines present focusing wavefront contributed from slits at different regions (I, II, or III). (**d**) (Left and middle) Simulated power intensity distributions of PL-C along the z- and y-axes. (Right) Focusing performance (FWHM and focal length divided by FWHM for the x- and y-axes) for each PL-C. The dashed line represents the diffraction limit of traditional objective lens at 316.5 nm (λ/2).

To design meta-aperture-based PLs for different applications, fundamental focusing properties of general PLs are examined first. Figure 1a illustrates the reference PL with circular nanoslit (PL- C) and our proposed meta-aperture-based PLs for modulation of focusing spot. The bowtie- nanoaperture (BN), L-shape-nanoaperture (LSN), and T-shape-nanoaperture (TSN) are chosen as the unit-apertures for PLs, which correspond to PL-B, PL-L, and PL-T, respectively. As shown in Fig. 1b, proper structural parameters for PL-C are selected by matching the focusing wavefront^16^, which can be expressed in terms of cylindrical coordinate as

1 $$\phi (r)=\frac{2\pi f}{\lambda }-\frac{2\pi \sqrt{{f}^{2}+{r}^{2}}}{\lambda }$$

Here, *f* is the focal length and λ is the working wavelength fixed at 633 nm. Circles, triangles, and hexagons represent the three cases studied without and with additional phase shift 2π (region II or III). The phase delays caused by different slit widths are calculated, and they could be extracted from the wavevectors of the metal-insulator-metal configuration. The analytical formula for the propagating wavevector *β*, which is crucial for the phase delay *βd*, can be written as^16–18^

2 $$\tanh (\sqrt{{\beta }^{2}-{k}_{o}^{2}{\varepsilon }_{{\rm{a}}{\rm{i}}{\rm{r}}}}w/2)=\frac{-{\varepsilon }_{{\rm{a}}{\rm{i}}{\rm{r}}}\sqrt{{\beta }^{2}-{k}_{o}^{2}{\varepsilon }_{{\rm{m}}}}}{{\varepsilon }_{{\rm{m}}}\sqrt{{\beta }^{2}-{k}_{o}^{2}{\varepsilon }_{{\rm{a}}{\rm{i}}{\rm{r}}}}}$$

Here, *k*_o_ represents the wavevector in free space, *w* is the slit width, and *ε*_air_ and *ε*_m_ are the permittivities in air and metal. Slit with specific width can be chosen at specific position by matching the phase delay condition as indicated in Fig. 1b. Different from those focusing surface plasmon waves at dielectric-metal interface^19,20^, meta-aperture-based PL focuses light above surface. Strong focusing spots with different focal lengths can be modulated by controlling the focusing wavefront, achieving long working distance operations. Figure 1c presents the power intensities and H_x_-field distributions in the y-z plane with dashed lines indicating focusing wavefronts. The black dashed line is formed by all the slits from different regions (I and II, or III). Figure 1d shows the focusing performance for each case of PL-C. The leftmost figure is the focusing profile along the z-axis and the middle plot reveals the focusing distribution along the y- axis. The rightmost plot compares the focusing properties of all cases. Here, we employ a focusing index defined as the focal length divided by the full maximum at half width (FWHM) to evaluate the focusing performance. As this index rises, the usability of focusing spot increases effectively, meaning long working distance with better resolution^21,22^. Among the cases of n = 2 (slits in region I), n = 3 (slits in region I, II), and n = 3 (slits in region I, III), the last one has the best focusing index 2 with subwavelength lateral resolution of 234 nm. From Fig. 1d, all focusing is in the near-field region because the focal lengths are smaller than one wavelength. The subwavelength focusing of PL-C is with the aid of evanescent waves. The diffraction limit is valid only for far-field due to loss of high spatial waves^23^. The black dashed line is the diffraction limit of traditional objective lens at our working wavelength, shown for comparison.

**Figure 2:**
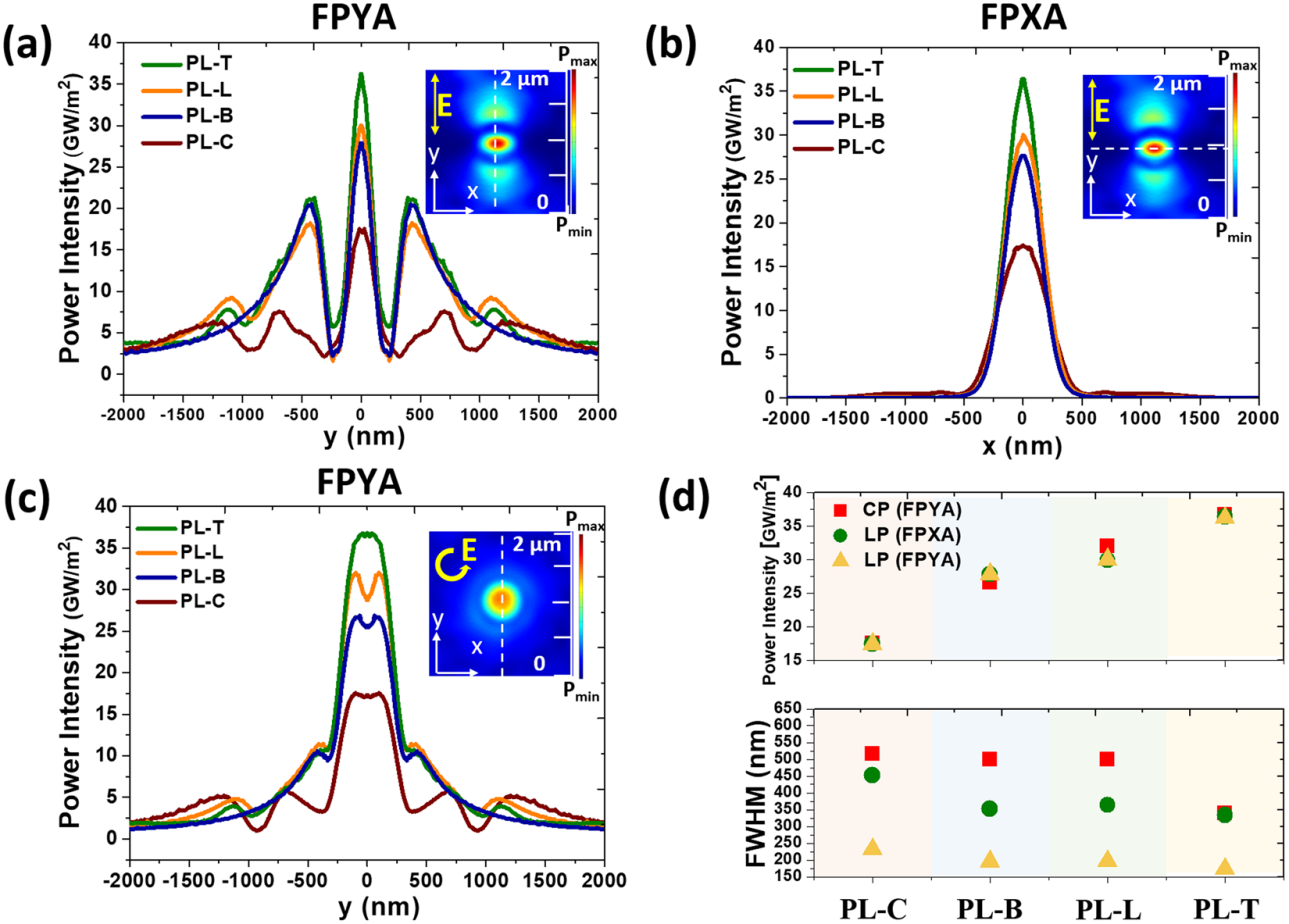
Simulated focusing power intensity profiles along (**a**) y- and (**b**) x-axes for PL-C, PL-B, PL-L, and PL-T under linear polarization (LP, parallel to the y-axis). (**c**) Simulated focusing power intensity profiles under right-handed circular polarization (CP) showing different shapes of focusing spots. (**d**) Comparison of power intensity and FWHM for PL-C, PL-B, PL-L, and PL-T.

Then, we employ BN, LSN, and TSN to replace the outer slits based on the case of n = 3 (slits in region I, III). The fundamental optical properties of nanoapertures are simulated and analyzed in Supplementary Fig. S1. The plasmon modes of these three nanoapertures are identified at the short, long resonance and working wavelengths. Figure 2a–c show simulated focusing profiles at the short, long resonance and working wavelengths. Figure 2a–c show simulated focusing profiles along the x and y-axes (FPXA and FPYA) under linear and circular polarizations. The insets are the power intensity distributions in the x-y plane. The focusing profiles reveal different beam shaping results (donut or spot shape) by different PLs under right-handed circular polarization. The Gaussian point spread function (PSF) for general imaging analysis is used^24,25^ to obtain the resolution. With the superposition of PSF, even the resolution of donut-shape profile can be calculated^25^. The radius of PSF at 1/e^2^ amplitude is taken as the line-width index *ω*_*D*_ proportional to FWHM^25^. Here, we show FWHM instead of *ω*_*D*_ for general comparison of resolution. In Fig. 2d, the focusing intensity and FWHM for FPXA or FPYA under linear polarization are presented. The FPYA of circular polarization is also shown to identify polarization effect. Here, the product of FWHM for x- and y-directions presented in terms of λ^2^ can be used as an index of focusing performance for the focal spot^26^. For the proposed meta-aperture-based PLs, PL-T exhibits the strongest focusing intensity with the narrowest subwavelength focal spot. The ultra-small simulated focusing spot achieves 0.14 λ^2^ under linear polarization, which exceeds many previous works^1,2,5,26^.

**Figure 3:**
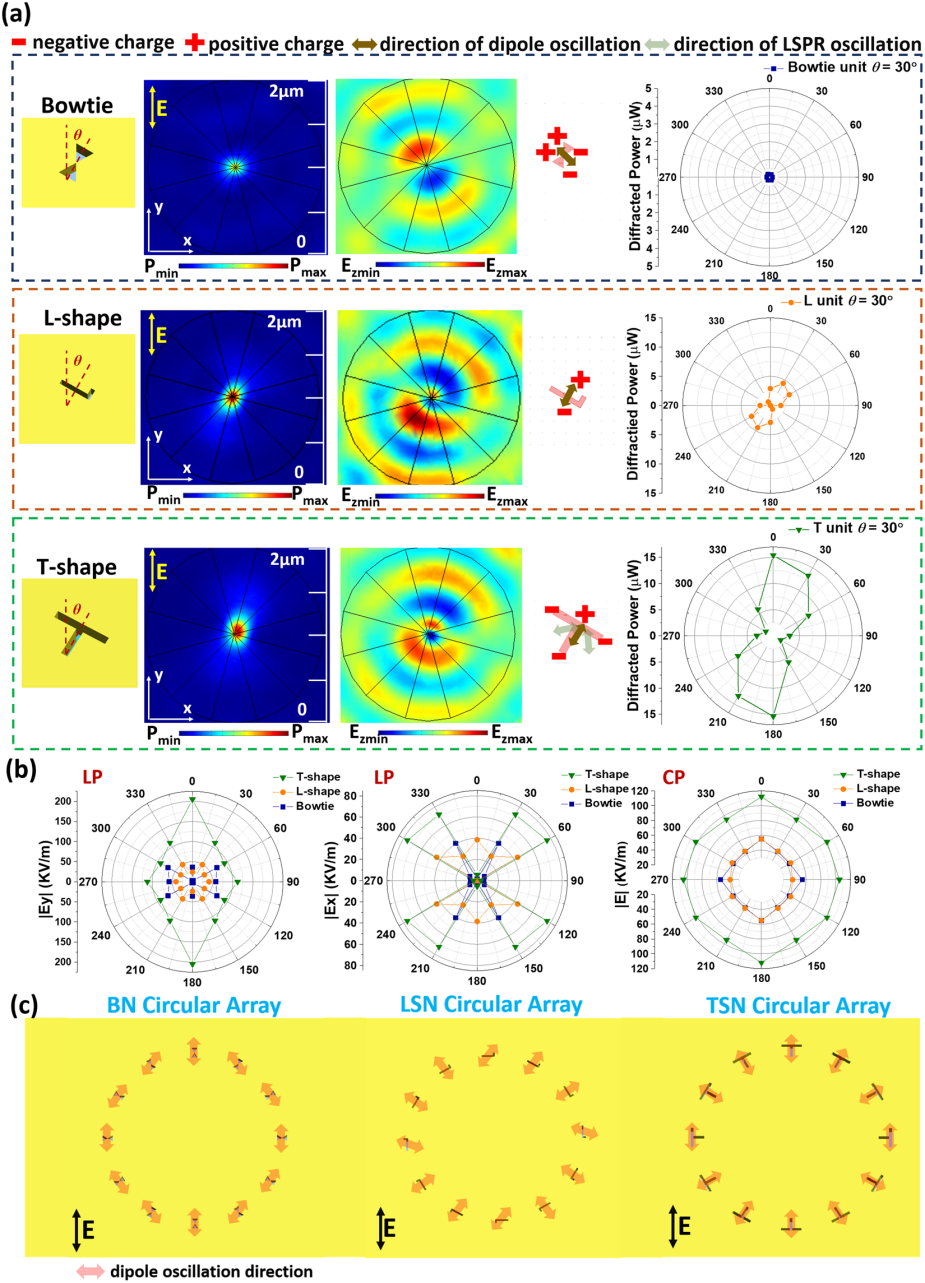
(**a**) Simulated power distributions, E_z_-fields, corresponding charge oscillations, and diffracted power patterns for BN, LSN, and TSN. The nanoapertures are rotated 30° with respect to the polarization. (**b**) Polar plot of the contributed |E_x_| and |E_y_| at focusing point is compared for BN, LSN, and TSN at each circular arranging position. Each point presents electric field amplitude contributed from a single nanoaperture with a specific *θ*. (**c**) Plots of dipole oscillation distribution for BN, LSN, and TSN circular array under linear polarization.

To analyze focusing performance, the diffraction characteristics of different nanoapertures are investigated in the near- and far-fields. The fundamental optical and diffraction properties of subwavelength nanoapertures have only been briefly described for simple circular and rectangular shapes^15^. The complex shape effect on diffraction of subwavelength nanoaperture has not been studied but has a great impact on our proposed PLs. In Fig. 3, we study the relationship between diffraction properties and rotation angle *θ*, where *θ* is the angle between the symmetry axis of nanoaperture and polarization direction. Then, contributions of diffraction from nanoapertures with different *θ* are examined to understand their influence on focusing resolution. Figure 3a shows the power intensity distributions, E_z_-fields, illustration of charge oscillations, and polar plots of diffracted power patterns for BN, LSN, and TSN at *θ* = 30°. For BN, the power intensity distribution reveals equally isotropic-angular-distributed diffraction. From the E_z_-field and polar plot of diffracted power, weak dipolar oscillation is observed. For LSN and TSN, the power intensity, E_z_-field, and diffracted power pattern show dipolar oscillation along the axis of *θ* = 30°. However, different charge distributions around the nanoaperture make a small difference of diffracted power distributions due to the shape effect. The charge distribution of Localized surface plasmon resonance (LSPR) mode of TSN is relative with its geometry, resulting the oscillation direction cross the corner, as indicated in Fig. 3a. From above analysis, BN provides equally isotropic-angular-distributed diffraction and acts as a source to generate scattering light in free space. LSN and TSN induce the axial dipolar oscillation, which can be seen as a highly directional point source.

The shape effect of nanoaperture with different *θ* on diffraction and dipole oscillation properties is thoroughly investigated in Supplementary Figs S2–S4 and summarized in Fig. 3b,c. Figure 3b provides the focusing contributions of nanoapertures with different *θ*. Each point is calculated by extracting selected electric-field component at focal point (z = 200 nm) contributed from single nanoaperture at a particular *θ*. According to the polar plots of |E_x_| and |E_y_| in Fig. 3b, we can calculate the ratio between E_x_ and E_y_ for each point. This ratio can be used to estimate the direction of local dipole oscillation denoted by φ = arg[E_y_/E_x_] = arg[tan(φ)] respect to the x-axis. The summation of |E_x_|^2^ + |E_y_|^2^ under linear polarization is proportional to the focusing intensity as calculated in Fig. 2. The summation of |E_x_|^2^ + |E_y_|^2^ of all *θ* for TSN is obviously larger than the others, which verifies the strongest focusing intensity of PL-T in Fig. 2d. For circular polarization, polar plots of |E| for BN, LSN, and TSN show rotational symmetry, resulting isotropic-polar-distributed focusing spot. The total |E|^2^ of all *θ* for TSN is also the largest among these nanoapertures, resulting the same impact on focusing as linear polarization.

Figure 3c shows the distributions of dipole oscillation direction for BN, LSN, and TSN circular arrays under linear polarization. For BN circular array, the azimuthal-like field distribution is obtained. For LSN and TSN circular arrays, the modulated field distribution in free space is similar to the radial polarized field. Furthermore, the radial polarization degree of output wavefront of TSN circular array is higher than that of LSN circular array. According to previous experiment verifications, the radial polarization can provide smaller lateral resolution compared to other polarizations^2,27,28^. Besides, the simulations of focusing properties of nanoaperture-based circular arrays in Supplementary Fig. S5 verify the model of dipole oscillation distribution.

These results confirm the characteristics of our proposed PLs under linear and circular polarizations, indicating the best focusing performance for TSN circular array.

### Experimental verification of focusing performance

The proposed meta-aperture-based PLs are fabricated and measured to verify their designed focusing performances. The Au thin film is deposited and patterned by electron-gun evaporator and focus ion beam (FIB). Detailed fabrication conditions and parameters are described in the method section. The upright measurement setup is built for bottom-up pumping method, which impinges normally onto the back surface of PL samples. Then, the scanning head mounted with fiber scans the x-y plane above the lens. With three-axis piezo scanner based on the technique of shear-force feedback, we measure the intensity distributions at different heights along the optical axis to find the focal plane with maximum intensity.

**Figure 4:**
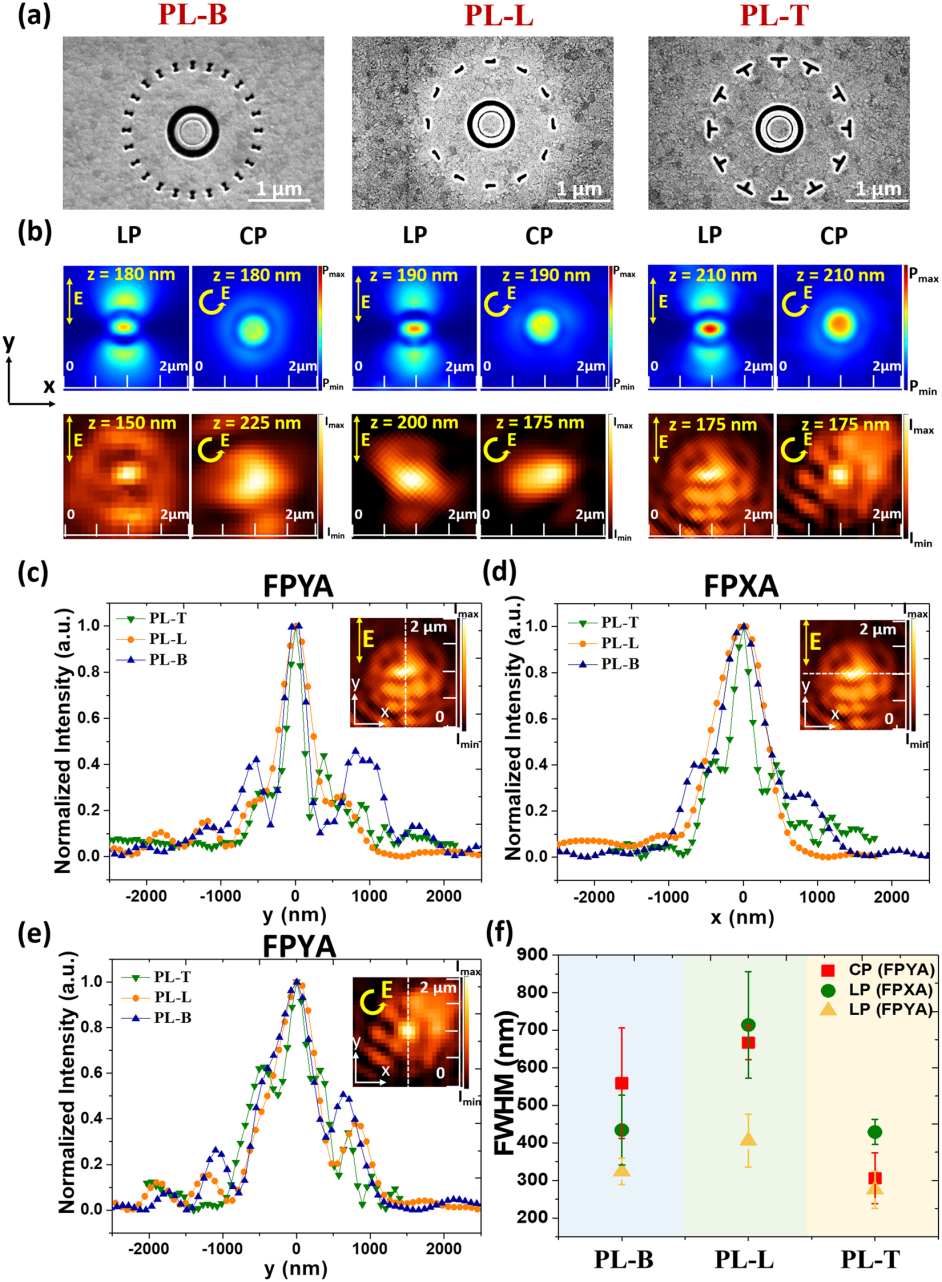
(**a**) SEM images of fabricated PL-B, PL-L, and PL-T. (**b**) Simulated and measured focusing profiles of PL-B, PL-L, and PL-T under different polarizations at each focal plane. Measured intensity distributions of focusing spot under linear polarization along the (**c**) y- and (**d**) x-axes. (**e**) Measured intensity distribution of focusing spot under circular polarization along the y-axis. (**f**) Summary of FWHM revealing the tuning range of each PL in experiments. PL-T exhibits the narrowest focusing spot indicating efficient modulation with this kind of nanoaperture.

Figure 4a shows the scanning electron microscope (SEM) images of PL-B, PL-L, and PL-T. Figure 4b presents simulated and measured focusing profiles at each focal plane under linear or circular polarization. The dipolar and circular power intensity distributions of focal spot for linear and circular polarizations are obtained. The FPXA and FPYA are shown in Fig. 4c–e. The best lateral resolution (FWHM) is found for PL-T with 0.35 λ in y direction under linear polarization. For circular polarization, PL-T also achieves the narrowest FWHM. The donut-shape focusing profile is not resolved in experiment because the peak-to-peak distance of donut is only 180 nm, close to the resolution of fiber. The resolution of fiber is determined by the aperture size, approximate 150 nm in our situation (details in the method section). All the measurement results are compared in Fig. 4f. Mean value is used for the center of location. The error bar is standard deviation to describe the variability of measured data. The FWHM in y direction under linear polarization is smaller than that in x direction for all cases. The FWHM for circular polarization is much larger than those for linear polarization as simulated in Fig. 2. The FWHM obtained in experiment is larger than that in simulation due to imperfect shape caused by fabrication. From Supplementary Fig. S6, increased radius of curvature broadens the lateral resolution significantly.

There are already many works for nanoslit-based PLs^12,13,17,29^ showing that small lateral resolution below 0.4 λ is hard to achieve owing to abbreviation and discontinuous focusing-parabolic wavefront causing incomplete constructive interference at focal point. By applying phase modulation of diffracted light from nanoapertures to overcome above challenges, lateral resolution smaller than 0.4 λ for PL-T is successfully obtained. The radial polarized wave has also been utilized for tight focusing. The ultra-small focal spot can achieve 0.16 λ^2^ without plasmonic structure. However, the excellent focusing is very restricted with using high numerical aperture^26,30^. In addition, many radial polarization converters are composed of only four half-wave plates, which is difficult for high purity of radial polarization. These problems can be solved by our meta-aperture-based PLs using linear or circular polarization. The simulated and measured smallest focusing spots for PL-T are 0.14 λ^2^ and 0.20 λ^2^.

Hence, PL-T reveals best focusing performance under both linear and circular polarizations. The focusing spot can be effectively shrunk by the modulation of diffracted wave from each nanoaperture. Furthermore, it exhibits extended focusing distance around four times longer than that of general superlens^31^.

### Application of nanoobject manipulation

For the past decades, the techniques of optical tweezers for nano/micro-particle manipulation have been widely studied. The force spectroscopy using optical tweezers is utilized to investigate biological molecules activities and DNA transcription^32^. Recently, the micro-rheology with mixed microfluid can support precise control of DNA transcription, which makes it more powerful in practice^33^. For this application, multiple trapping is needed but has seldom been discussed. A designed aperture acting as a plasmonic nanocavity can provide multiple-nanoparticles manipulation by strong near-field of plasmonic aperture mode^34^. However, this kind of trapping is limited near the surface. Here, we use the proposed PL-T to achieve not only multiple trapping but also long-distance operation, that is, above the surface, to increase the practicability.

**Figure 5:**
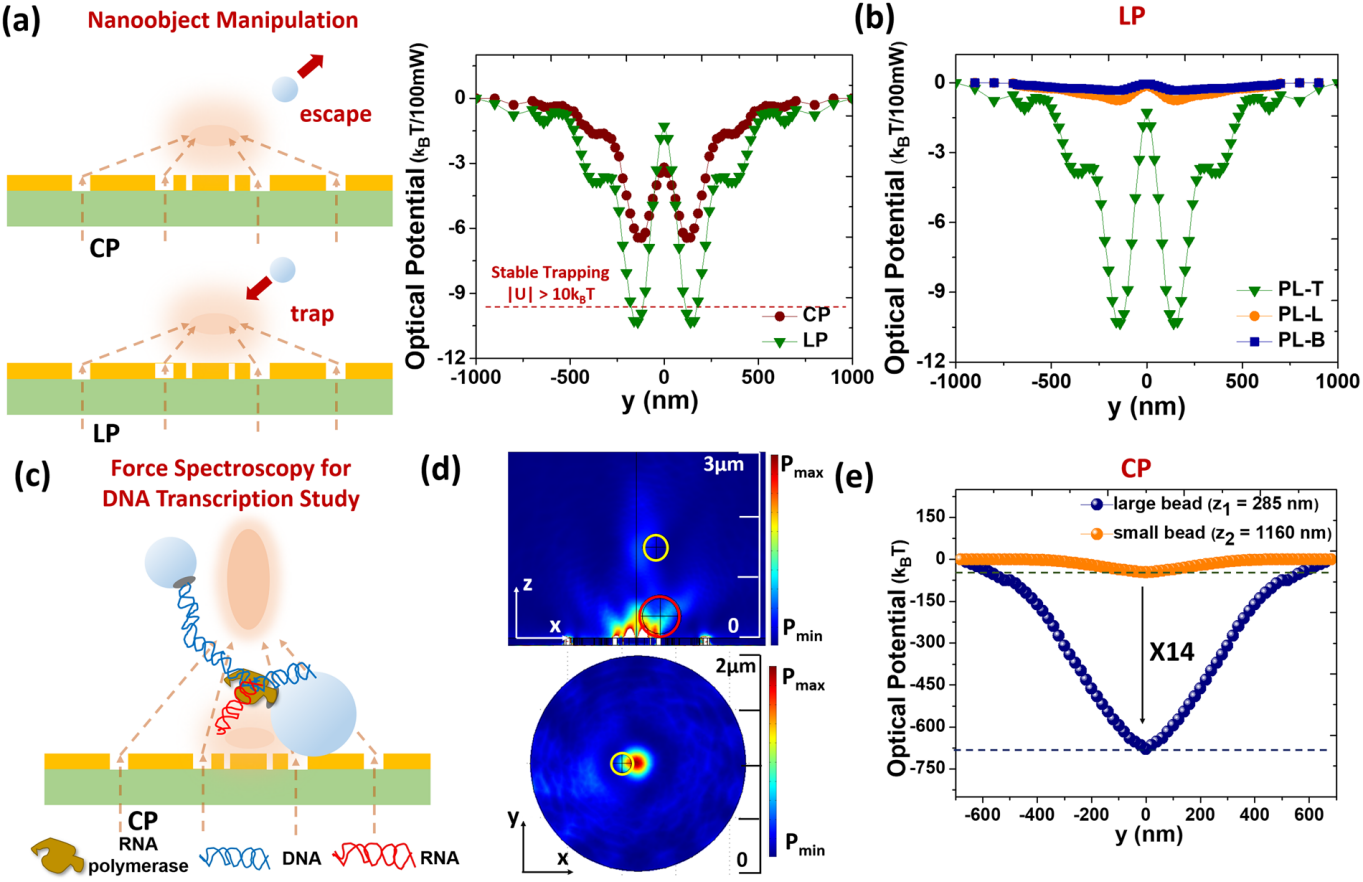
(**a**) Illustration of nanoobject manipulation by dynamic control of polarization. PL-T under linearly polarized illumination can stably trap 100 nm nanoparticle but not under circularly polarized condition. (**b**) Simulated optical potential produced by PL-B, PL-L, and PL-T. Only PL-T with efficiently modulated focal performance can provide sufficient trapping potential. (**c**) Illustration of multiple particle manipulation of PL-T as force spectroscopy for studying DNA transcription. (**d**) Simulated power intensity distributions of PL-T under circular polarization show two focal spots along the z-axis. (**e**) Simulated optical potential distribution at each focal plane showing the trapping ability of PL-T away from the surface (>1 μm). The optical potential experienced by the large bead is around 14 times deeper than that by the small bead.

The trapping force and optical potential are calculated using Maxwell stress tensor^35^. A 100 nm-diameter polystyrene sphere is selected as trapping target with refractive index of 1.6. The refractive index of water environment is 1.33. Figure 5a shows the dynamic operations of trapping and releasing nanoparticle by switching the polarization. By the estimation of simulation, the requirement for stable trapping (|U| > 10k_B_T) can be fulfilled for PL-T under linear polarization but not under circular polarization for pump power of 100 mW. Among these PLs, only PL-T exhibits enough optical potential to provide trapping ability under linear polarization, as shown in Fig. 5b. Surmounting the restriction near the surface, we successfully demonstrate trapping of subwavelength nanoobject 200 nm above the surface.

Figure 5c depicts the application of force spectroscopy for DNA transcription requiring multiple trapping. Since PL-T under circular polarization possesses multiple focusing spots, it can be used to trap the submicron binding beads linked by the DNA strain. The total focusing wavefront from regions I and III in water environment is extended to a higher height and an additional focusing spot appears closer to the surface (only from region I), resulting multiple focusing spots. By optical trapping for monitor transcriptional elongation with near-base-pair precision, the cleavage and backtracking events between RNA and RNA polymerase (RNAP) can be detected^36^. Figure 5d shows the power intensity distributions of two-bead trapping in the x-z plane and the high focal plane. The yellow and red circles represent the two beads with radii of 175 and 275 nm respectively. For the process of DNA transcription, the large bead should be trapped close to the surface and the small bead can be trapped at the high focal plane. The large bead experiences optical potential around 10 times deeper than the small bead does, which can provide relative stability for the large bead in the DNA transcription^36^. Figure 5e shows y-directional distribution of optical potential for small and large beads at each focal plane. The parameters z_1_ and z_2_ for large and small beads are 285 and 1160 nm, defined as the distance from the center of bead to surface. The sufficient optical potential enables trapping at long distance (>1 μm) by plasmonic nanostructure compared to near- field supported device^31^. This method provides a simplified way to study DNA transcription by utilizing only one pumping beam instead of two trapping beams^36^.

## Conclusion

The meta-aperture-based PLs are designed, fabricated, and demonstrated for efficient subwavelength focusing modulation under linear and circular polarizations. The BN, LSN, and TSN are proposed for diffraction tuning, acting as meta-units to shape the focusing profiles for PL-B, PL-L, and PL-T with circularly arranged nanoapertures. The shape-dependent diffraction properties are investigated, which can be used to explain the focusing performance. BN generates isotropic-angular-distributed diffraction. LSN and TSN perform as highly directional point sources of diffraction. The TSN circular array provides most efficient focusing modulation under both linear and circular polarizations. Hence, PL-T achieves ultra-small focusing spot of 0.14 λ^2^ and 0.20 λ^2^ (λ = 633 nm) for simulation and experiment respectively, especially under linear polarization. This performance is better than most focusing devices under linear polarization, even exceeding the performance of those using radial polarization with general numerical aperture objective lens. Our proposed PL-T exhibits not only superior resolution but also extended focusing distance around four times longer than that of general superlens, increasing its usability. The dynamic trapping and releasing of 100 nm subwavelength nanoparticle 200 nm away from the surface is realized by PL-T. PL-T can also provide two strong focal spots for multiple-object manipulation, which can be used for DNA transcription study. PLs with different designs of nanoaperture can serve as a platform for various applications, which is important for on-chip nanotechnologies development.

## Methods

### Numerical method

Finite element method (FEM) calculations were performed to simulate the focusing properties of meta-aperture-based PLs. The dielectric function of Au is taken from a previous work by evaporation method^37^. Transverse magnetic (TM)-polarized planer wave is setup (electric-field *E* parallel to the y-axis) for linear excitation. For circular polarization, the phase difference of E_x_ and E_y_ is fixed at π/2. Then, the power intensity distributions in the x-y plane were obtained and different directional resolutions can be analyzed.

### Fabrication of meta-aperture-based PL

The fabrication process is simple and can be achieved in two steps. The glass was selected as a substrate and the gold film was deposited by the electron-gun evaporation (EBX-8C, ULVAC). Slow evaporation rate is set at 0.3 Å/s to ensure the high quality of film with less roughness. Then, the patterns of various PLs were sketched by the focus ion beam (FIB) milling technique (Ga + ion, Helios NanoLab 600i dual beam, FEI). The fine structure was realized by the operation at 30 kV and 10 pA beam current.

### Measurement of near-fields

The near-field scanning optical microscope (NSOM, NT-MDT NTEGRA Solaris) was used to image the near-field distributions at different heights from the surface to the focal plane. The home-made fiber is mounted on a tuning fork, whose aperture is around 150 nm and is coated with 130-nm-thick silver film on the outer surface of the taper of the fiber for ensuring high collection efficiency^38^. Besides, the shear-force feedback is employed with electrically biased voltage for scanning the images at different heights. In addition, the white light laser (SuperK EXTREME supercontinuum lasers, NKT) used as a source, whose wavelength can be selected by the filter, was incident on the back-side of the sample and transmitted through PL. Finally, the near-field is sampled by the probe and transmitted through the fiber into the photomultiplier tube for signal detection.

## Electronic supplementary material

Supplementary Information
