# Supplementary material for: Efficient modulation of subwavelength focusing via meta-aperture-based plasmonic lens for multifunction applications
Source: Sci Rep. 2018 Sep 11;8:13648. doi: 10.1038/s41598-018-31860-1 (PMC6134010; doi:10.1038/s41598-018-31860-1)
Supplement: Supplementary file 1 — Supplementary Information [file 41598_2018_31860_MOESM1_ESM.docx]

Supplementary Information

**Efficient Modulation of Subwavelength Focusing via Meta-Aperture-Based Plasmonic Lens for Multifunction Applications**

Kai-Hao Chang^1^, Yen-Chun Chen^2^, Wen-Hao Chang^2^, and Po-Tsung Lee^1*^

^1^Department of Photonics, College of Electrical and Computer Engineering, National Chiao Tung University, Hsinchu 300, Taiwan

^2^Department of Electrophysics, College of Science, National Chiao Tung University, Hsinchu 300, Taiwan

* Corresponding Author: Po-Tsung Lee, E-mail: [potsung@mail.nctu.edu.tw](mailto:potsung@mail.nctu.edu.tw)

**S1 Optical properties of subwavelength nanoaperture**

The fundamental optical properties of nanoapertures are simulated and analyzed in Fig. S1. The normalized transmittance spectra excluding the aperture-size effect^1^ are calculated for the proposed bowtie-nanoaperture (BN), L-shape-nanoaperture (LSN), and T-shape-nanoaperture (TSN). Figure S1a reveals two resonance peaks for both linear and circular polarizations and their corresponding electric field distributions in the x-y and y-z planes are provided in Fig. S1b. Form the electric field distributions in the y-z plane under linear polarization, the resonance modes at λ = 840 nm for LSN and 820 nm for TSN are identified as the second-order plasmon resonance (SPR) mode^2^ distributed within the full nanoaperture. For the case of λ = 570 nm for LSN and 560 nm for TSN under linear polarization, the mode is localized at the top edge of nanoaperture, known as single-surface first-order plasmon resonance (SFPR) mode^2^. For BN, weak coupling at λ = 590 and 780 nm is observed. Under circular polarization, more uniform mode profiles are obtained for all cases. The Fabry-Peröt-like distributions of electric-field in the y-z plane are found in SPR at λ = 840 nm for LSN and 820 nm for TSN. The other resonances of plasmon mode are found at λ = 580 nm for LSN and 570 nm for TSN. In addition, the field distributions of plasmon modes at our experimental working wavelength λ = 633 nm are similar to those of resonance modes at the shorter wavelength. These plasmon modes at λ = 633 nm can be identified as the SFPR mode by simulation. All nanoapertures are design with specific dimension for matching the same resonance wavelength, which can be examined in the labeled figures (λ).


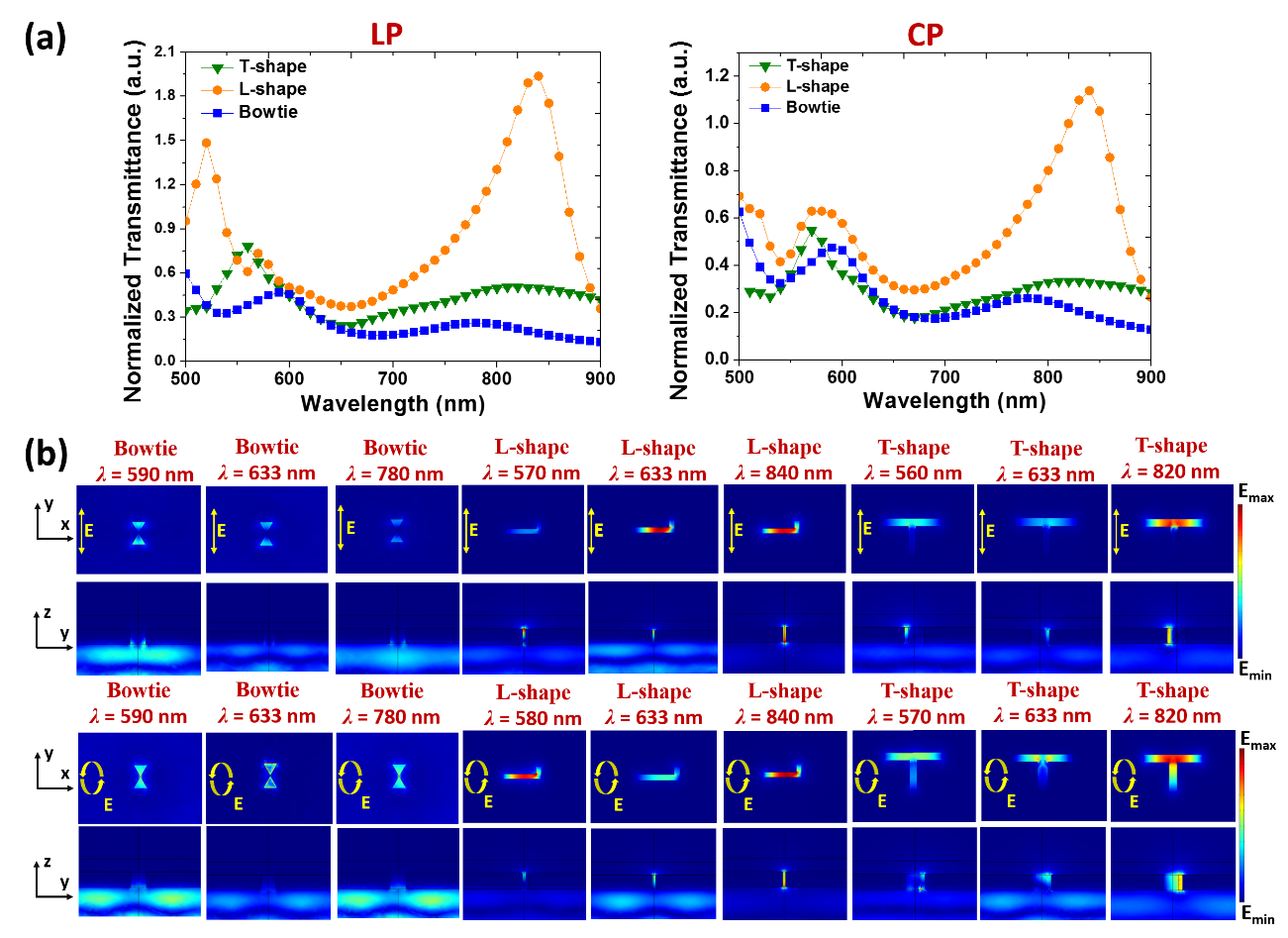


**Figure S1.** **Optical properties of the proposed nanoapertures under linear and circular polarizations.** (a) Transmission spectra for BN, LSN, and TSN. Two plasmon resonance modes are observed for both polarizations. (b) Electric field distributions in the x-y and y-z planes showing the plasmon modes at the short resonance, working, and long resonance wavelengths.

**S2** **Shape effect on dipole oscillation of subwavelength nanoaperture**

The relation between shape-dependent diffraction properties and local charge oscillation is verified in Fig. 3 in the main text. The power intensity distribution, E_z_ plot, and illustration of charge distribution under linear polarization are shown in Figs. S2-4 to describe the diffraction properties for BN, LSN, and TSN with rotation angle *θ* from 0° to 180°. The full angle analysis can be obtained by the symmetry imaging. To further verify the result of dipole oscillation model in Fig. 3c (in the main text), the focusing properties of nanoaperture-based circular array are simulated. The focusing profiles for BN, LSN, and TSN circular arrays are compared under linear and circular polarizations in Fig. S5, which shows good agreement with the focusing performance in Fig. 2. The TSN circular array provides the narrowest focusing FWHM under both polarizations.


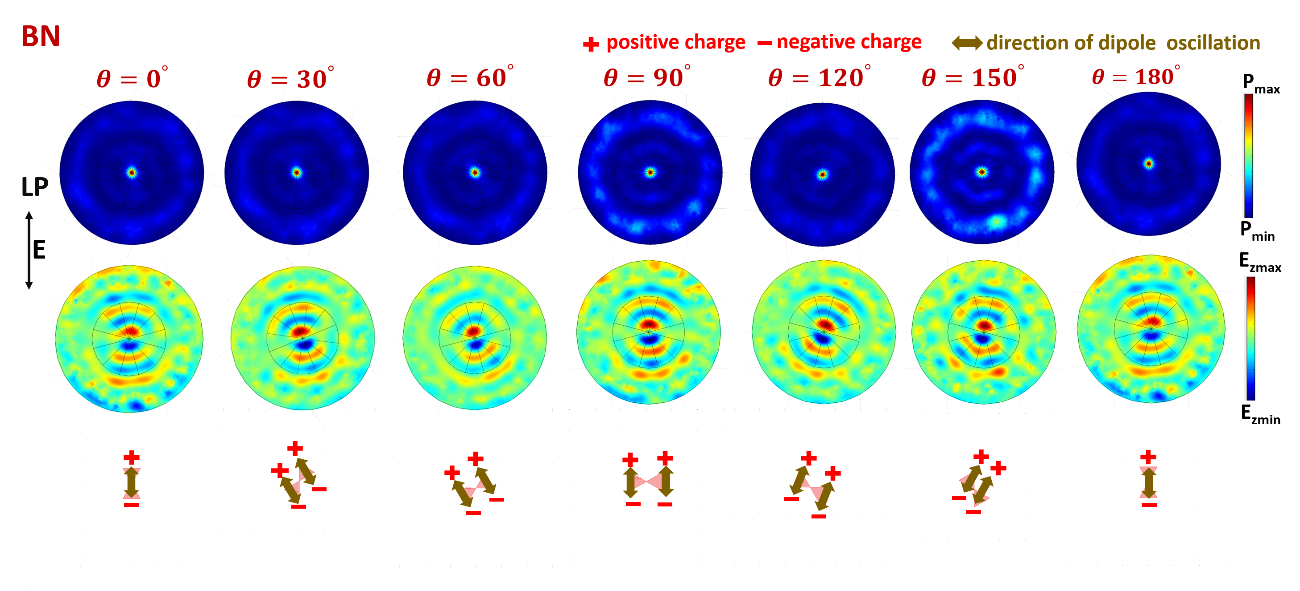


**Figure S2. Dipole oscillation of BN for different rotation angles.**

The power intensity distributions, E_z_ plots, and charge oscillation of BN with different rotation angles *θ* from 0° to 180° under linear polarization.


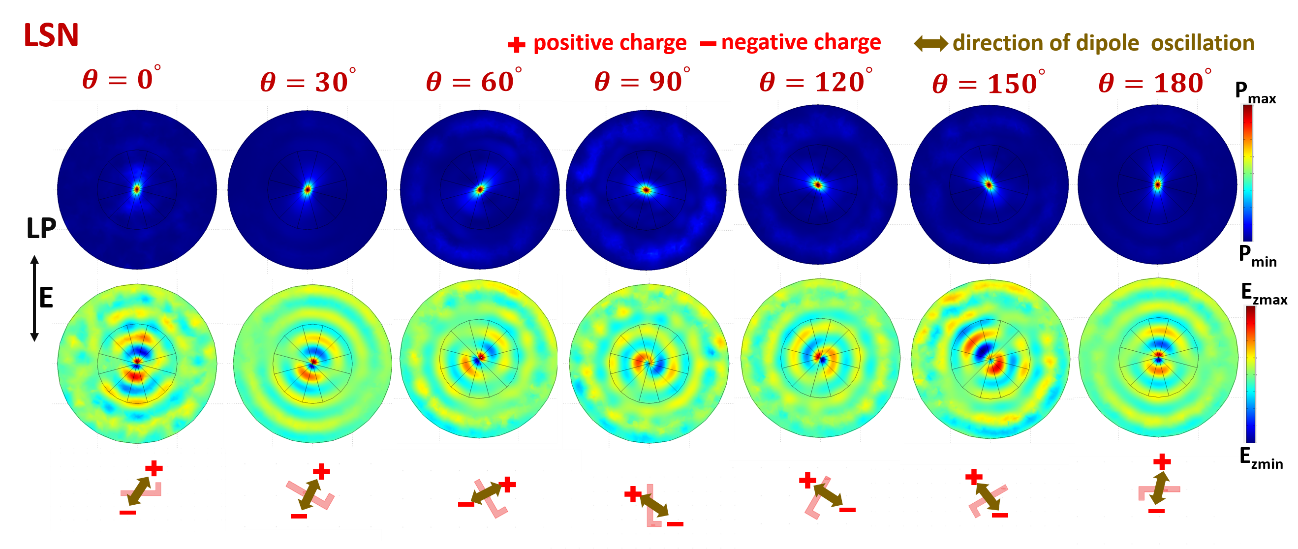


**Figure S3. Dipole oscillation of LSN for different rotation angles.**

The power intensity distributions, E_z_ plots, and charge oscillation of LSN with different rotation angles *θ* from 0° to 180° under linear polarization.


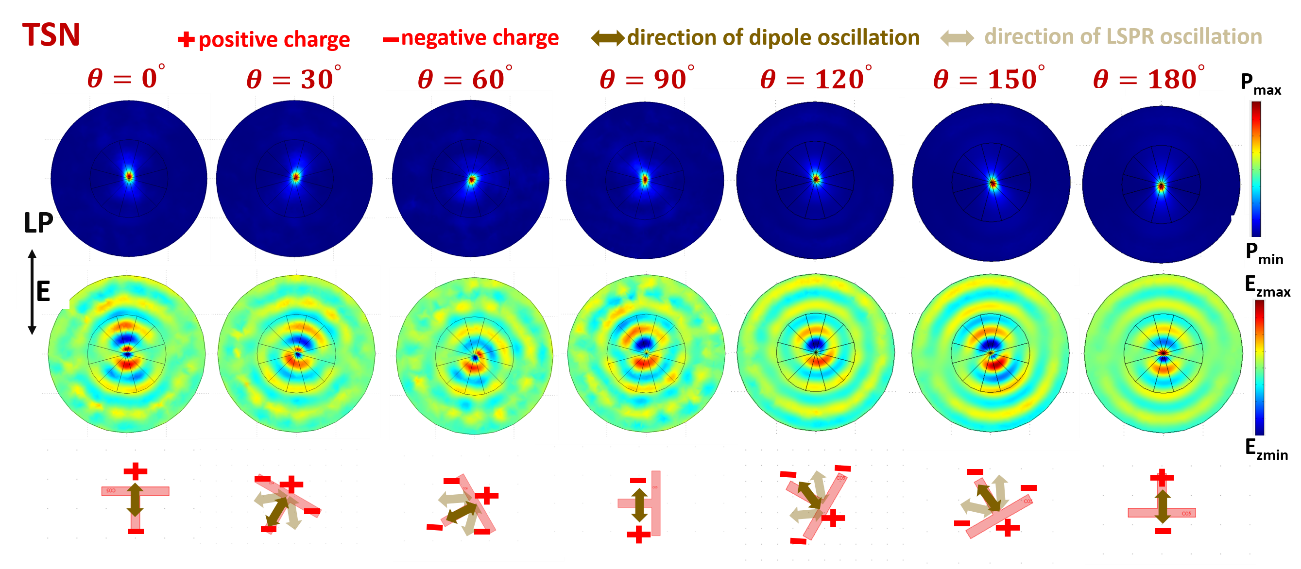


**Figure S4. Dipole oscillation of TSN for different rotation angles.**

The power intensity distributions, E_z_ plots, and charge oscillation of BN with different rotation angles *θ* from 0° to 180° under linear polarization. The transparent arrow indicates the direction of LSPR oscillation.


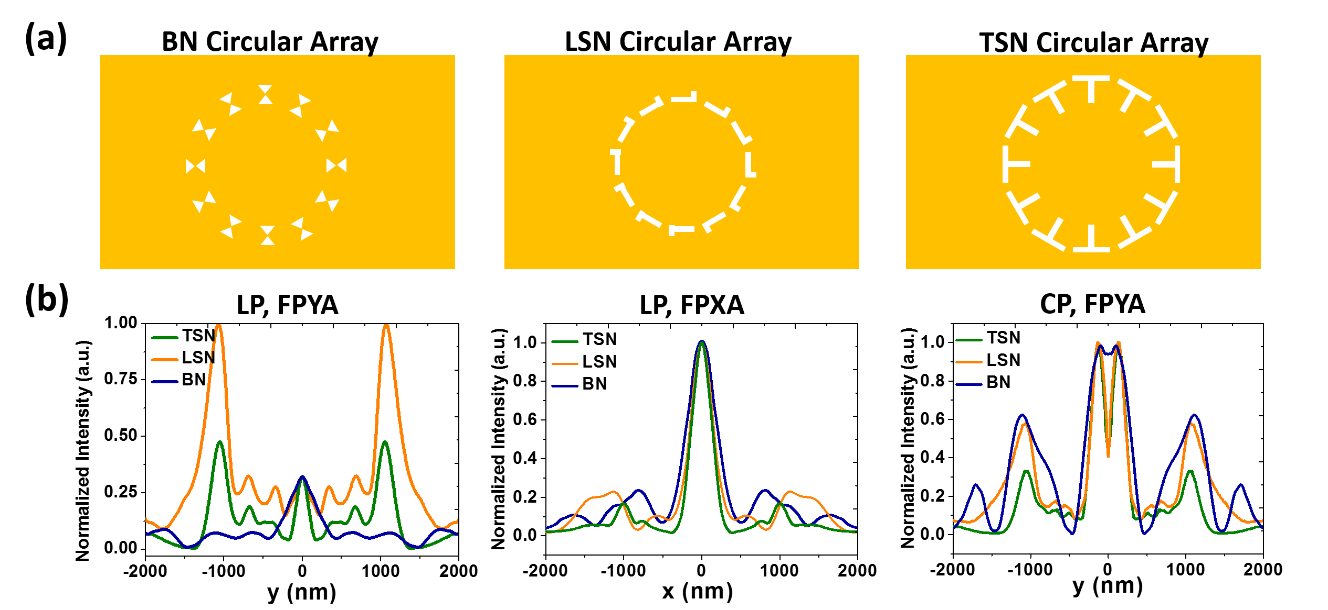


**Figure S5. Focusing properties of nanoaperture-based circular arrays.**

(a) Illustration of circular nanoaperture arrangement for BN, LSN and TSN circular arrays. (b) Focusing profiles along the x and y-axes (FPXA and FPYA) of BN, LSN and TSN circular arrays under linear and circular polarizations.

**S3 Morphology effect on focusing**

The nonperfect shape after FIB milling produces additional scattering resulting in broadened focusing spot as illustrated in Fig. S6a. The arrow indicates the radius of curvature *r* of nanoslit and nanoaperture for PL-T. Figure S6b shows the broadened donut-shape focusing profiles for different *r* under circular polarization. The lateral resolution enlarges from 335 to 650 nm as *r* varies from 0 to 90 nm. Obvious increment of FWHM is observed when *r* changes from 30 to 60 nm due to increased scattering near nanoslit, as shown in the power intensity distribution in Fig. S6a.


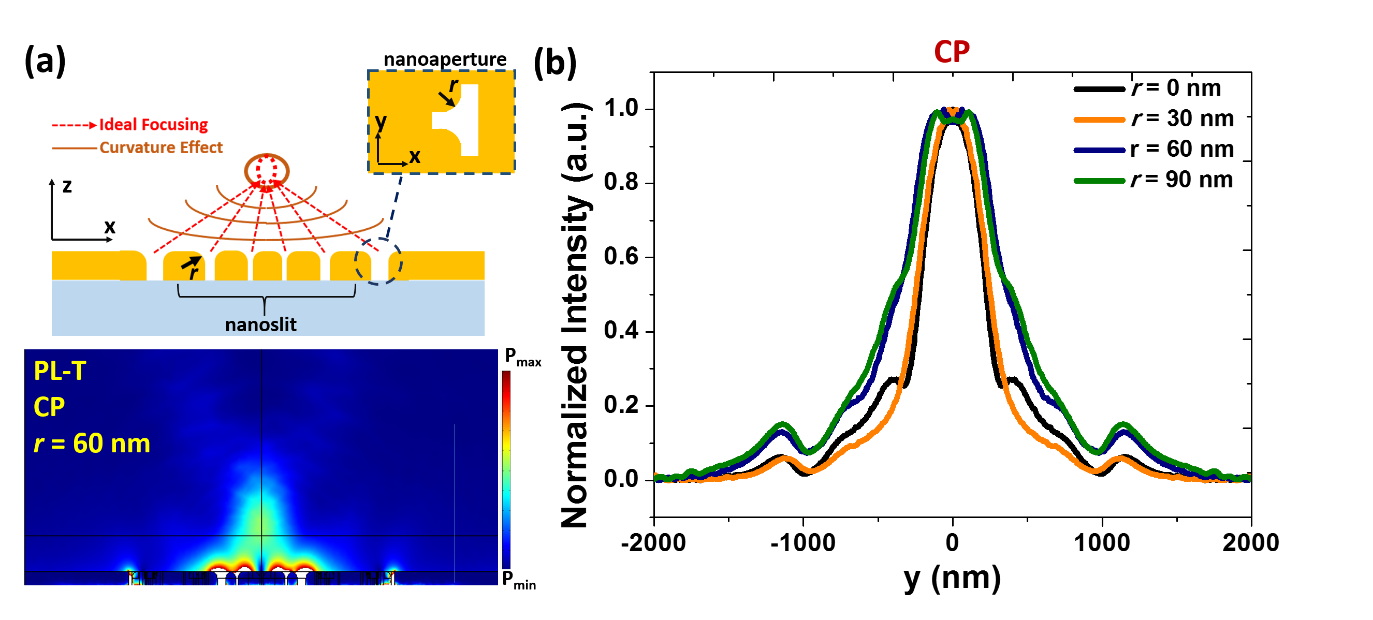


**Figure S6. Effect of curvature radius on focusing performance.**

(a) Illustration of broadened focusing spot caused by nonperfect shape and the power intensity distribution of PL-T with *r* = 60 nm under circular polarization. (b) Normalized power intensity distributions for different *r*.

**Supplemental References**

1. Chang, S. H., Gray, S. K., & Schatz, G. C. Surface plasmon generation and light transmission by isolated nanoholes and arrays of nanoholes in thin metal films. *Opt. Express* **13**, 3150–3165 (2005).
2. Yang, J., Hu, C., Wen, Q., Zhao, C., & Zhang, J. Coupling between surface plasmon polaritons and transverse electric polarized light via L-shaped nano-apertures. *Opt. Lett.* **40**, 978–981 (2015).
